# Genome-wide DNA methylation analysis in jejunum of *Sus scrofa* with intrauterine growth restriction

**DOI:** 10.1007/s00438-018-1422-9

**Published:** 2018-02-01

**Authors:** Yue Hu, Liang Hu, Desheng Gong, Hanlin Lu, Yue Xuan, Ru Wang, De Wu, Daiwen Chen, Keying Zhang, Fei Gao, Lianqiang Che

**Affiliations:** 1 Institute of Animal Nutrition, Sichuan Agricultural University, Ya’an, 625014 Sichuan China; 2 Genome Analysis Laboratory of the Ministry of Agriculture, Agricultural Genomics Institute at Shenzhen, Chinese Academy of Agricultural Sciences, Shenzhen, 518120 China

**Keywords:** Intrauterine growth restriction (IUGR), Fetal growth, Epigenetics, Intestinal function

## Abstract

Intrauterine growth restriction (IUGR) may elicit a series of postnatal body developmental and metabolic diseases due to their impaired growth and development in the mammalian embryo/fetus during pregnancy. In the present study, we hypothesized that IUGR may lead to abnormally regulated DNA methylation in the intestine, causing intestinal dysfunctions. We applied reduced representation bisulfite sequencing (RRBS) technology to study the jejunum tissues from four newborn IUGR piglets and their normal body weight (NBW) littermates. The results revealed extensively regional DNA methylation changes between IUGR/NBW pairs from different gilts, affecting dozens of genes. Hiseq-based bisulfite sequencing PCR (Hiseq-BSP) was used for validations of 19 genes with epigenetic abnormality, confirming three genes (AIFM1, MTMR1, and TWIST2) in extra samples. Furthermore, integrated analysis of these 19 genes with proteome data indicated that there were three main genes (BCAP31, IRAK1, and AIFM1) interacting with important immunity- or metabolism-related proteins, which could explain the potential intestinal dysfunctions of IUGR piglets. We conclude that IUGR can lead to disparate DNA methylation in the intestine and these changes may affect several important biological processes such as cell apoptosis, cell differentiation, and immunity, which provides more clues linking IUGR and its long-term complications.

**Electronic supplementary material:**

The online version of this article (10.1007/s00438-018-1422-9) contains supplementary material, which is available to authorized users.

## Introduction

Intrauterine growth restriction (IUGR) is defined as impaired growth and development of the mammalian embryo/fetus or its organs during pregnancy. IUGR affects about 5% of human neonates, with a high risk of perinatal morbidity and mortality (Sharma et al. 2016). It has been reported that there are long-term complications of IUGR offspring including increased risk of developing metabolic syndrome, cardiovascular disease, and type II diabetes in adulthood (Salam et al. 2014). Furthermore, IUGR neonates have been shown to display extensive dysfunction of the gastrointestinal (GI) tract, including poor digestion and absorption of nutrients (Karagianni et al. 2010; He et al. 2011; Mickiewicz et al. 2012), enhanced cell apoptosis (Baserga et al. 2004), and impaired barrier function (Fança-Berthon et al. 2009). The changed transcriptomic and proteomic profiles indicated that IUGR intestine had cellular signaling defects, redox imbalance, and enhanced proteolysis (Wang et al. 2008; D’Inca et al. 2010), suggesting that certain molecular mechanisms are involved in the intestinal dysfunction of IUGR neonates.

As an important type of epigenetic modifications, DNA methylation plays an essential role in transcriptomic regulation (El Taghdouini et al. 2015; Davies et al. 2017). In addition, evidences on nutritional epigenetics suggest that nutrients can modify DNA methylation (Lillycrop et al. 2005; Anderson et al. 2012; Murdoch et al. 2016). Due to the impaired placental functions, the fetus is exposed to intrauterine environment with limited nutrients that may ultimately affect the intestinal epigenome. It has been reported that IUGR in monozygotic twins is associated with the abnormal methylation in placental genes involved in lipid metabolism and transcriptional regulation as well as in cadherin and Wnt signaling pathways (Roifman et al. 2016). In addition, growth restricted neonates also have been proved to possess distinct DNA methylation profiles in placenta and cord blood at birth, which were hypothesized to predispose to adult disease (Hillman et al. 2015).

In the present study, we hypothesized that IUGR fetal intestines had abnormal DNA methylation, which might be carried forward to postnatal period, resulting in the abnormal intestinal gene expression and related impairments in intestinal development and function. We established a pig model of IUGR according to the standard that piglets were at least 1.5 SD lower birth weight compared to their NBW littermates (Che et al. 2010). Contrast with the rodent model (Reamon-Buettner et al. 2014), pig could be a better animal model to study IUGR because of its high similarities in body metabolism and function, as well as prenatal and postnatal development of the gastrointestinal tract with humans (Ferenc et al. 2014; Jiang and Sangild 2014). RRBS, a genome-scale, relatively low-cost method for pig DNA methylome analysis, was applied to study the DNA methylation changes in the jejunum tissues of IUGR piglets. We found the alterations of differentially methylated regions (DMRs) in IUGR piglets compared to their NBW littermates, with three related key genes (AIFM1, MTMR1, and TWIST2) which were successfully validated in independent sample sets. In addition, integrative analyses with a proteome study revealed that three DMR-related genes (DMRGs) (BCAP31, IRAK1, and AIFM1) could interact with important immunity- or metabolism-related proteins. In summary, our data support the hypothesis that IUGR could lead to DNA methylation changes in the intestinal tissues, which may modulate the expression of genes related to cell apoptosis, differentiation, and immunity.

## Materials and methods

### Piglet model and tissue collection

The pregnant gilts (Landrace genotype, *n* = 4) were fed with corn and soybean meal-based diet (2.5 kg/day), with free access to drinking water. Four litters of piglets (IUGR/NBW pair 1–4) were delivered from gilts at term (114 days of gestation). According to our previous study (Che et al. 2010), pigs with a birth weight near the mean birth weight (± 0.5 SD) were identified as NBW, whereas pigs at least 1.5 SD lower birth weight were defined as IUGR, and then, both NBW and IUGR pigs were killed by jugular puncture after anesthesia with an intravenous injection of sodium pentobarbital (15 mg/KG BW). Small intestinal length was measured on an ice-cooled plate and divided into three equal segments designated proximal, middle, and distal SI. Samples from each intestinal region were opened along its length for measurement of intestinal circumference and wet weight. The experiments followed the actual law of animal protection and were approved by the Animal Care and Use Committee of the Sichuan Agricultural University.

### Plasma urea and amino acid analysis

As our previous study (Peng et al. 2016), plasma urea was measured using a biochemistry analyzer (Beckman CX4) according to the manufacturer’s instructions. For the plasma amino acids (AA) contents, briefly, 1 ml of plasma and 2.5 ml of 7.5% trichloroacetic acid were mixed thoroughly and centrifuged at 12,000×*g* at 4 °C for 15 min. The supernatant was analyzed for amino acids using an auto amino acid analyzer (L-8800; Hitachi, Tokyo, Japan).

### Quantitative real-time PCR

The total RNAs of 8 jejunum samples (middle portion) from IUGR/NBW pair 1–4 were extracted by the RNeasy Mini kit (QIAGEN) following the manufacturer’s instructions. Qualification of RNA samples were carried out by agarose gel electrophoresis and the concentration were detected by Qubit 2.0. The 500 ng of total RNA was reversely transcribed using oligo (dT) 12–18 primer with Superscript II reverse transcriptase (Invitrogen) according to the manufacturer’s instructions. Primers for real-time quantitative PCR (RT-qPCR) were designed using Primer 5 software and listed in S2 Table. Real-time qPCR analysis was performed on ABI Prism 7700 (Applied Biosystems, Tokyo, Japan) using SYBR Green real-time PCR master mix (Toyobo Co., Japan). Relative expression levels of objective mRNAs were calculated using the ∆∆Ct method and normalized to GAPDH. All data were presented as the mean values ± SE. Comparisons were made using the Student’s *t* test and a two-sided *P* value < 0.05 was considered to indicate statistical significance.

### The RRBS library construction

The eight jejunum samples (the middle portion) from IUGR/NBW pair 1–4 were selected for RRBS analysis. First, extractions of genomic DNA were completed by DNeasy Blood & Tissue Kit (QIAGEN) according to manufacturer’s instructions. Qualifications were detected by agarose gel electrophoresis and the concentrations were detected by Qubit 2.0. RRBS library construction was performed as previously described by Wang et al. (Wang et al. 2012). Briefly, 4 µg of genomic DNA were digested with 100 U of *Msp* I enzymes (NEB) at 37 °C for 16 hs, followed by blunt-ending, dATP addition, and methylated-adapter ligation. To obtain DNA fractions of 40–120 and 120–220 bp of *Msp* I-digested products, two ranges of 160–240 and 240–340 bp adapter ligated fractions were excised from a 2% agarose gel, respectively. Bisulfite conversion was conducted by ZYMO EZ DNA Methylation-Gold Kit™ (ZYMO) following the manufacturer’s instructions. By bisulfite treatment, a methylated cytosine maintains as “C”, while a non-methylated cytosine is transferred to “U” and final “T” after polymerase chain reaction (PCR) amplification. For cytosine sites in CpG and non-CpG context, we first calculated the ratio of “C” reads to the total reads to get a global methylation level of CpG and non-CpG context, respectively. The final libraries were generated by PCR amplification using JumpStartTM Taq DNA Polymerase (Sigma) (11 cycles for 160–240 bp and 13 cycles for 240–340 bp). RRBS libraries were then analyzed by Agilent 2100 Bioanalyzer (Agilent Technologies) and quantified by qPCR.

### RRBS sequencing and data processing

The RRBS libraries were sequenced using Illumina Hiseq2000 analyzer with paired end reads of 50 bp read length (PE50) according to the manufacturer’s instructions. Raw sequencing data were processed by the Illumina base-calling pipeline. Adapter contamination and low-quality reads that contained more than 30%’N’s or over 10% of the sequence with low-quality value (quality value < 20) per read were omitted from the data analysis. The pig reference genome (*Sus scrofa*10.2) by Swine Genome Sequencing Consortium (SGSC) was downloaded, and then, these clean reads were aligned to the pig reference genome in an unbiased way for bisulfite sequencing data, as published in the previous studies (Li et al. 2010; Wang et al. 2013): (1) all the observed cytosines were replaced by thymines and the guanines were replaced by adenosines in silico, forming two “alignment form” references; (2) observed cytosines on the forward read of each read pair were replaced by thymines, and observed guanines on the reverse read of each read pair were replaced by adenosines, in silico; (3) then, the “alignment form” reads were mapped to the “alignment form” reference using SOAPaligner (version 2.01) (http://soap.genomics.org.cn/) (Li et al. 2009). The uniquely aligned reads that contained *MspI* digestion site at the ends were used and the first two bases (*MspI*) on the 50 end of the reverse reads that were filled in during the end-repair were masked for further analysis.

### Identification of differential modification regions (DMRs) and enrichment analysis of DMR-related genes (DMRGs)

Methylation level of individual cytosines was defined as the ratio of methylated “C” reads to total sequenced reads as previously described (Gao et al. 2014). First, commonly covered CpG sites with sequencing depth ≥ 5 between IUGR and its NBW littermate were selected as candidate sites. Methylation level of individual cytosine can then be defined as the ratio of “C” counts to total counts of “C” and “T” in the sequenced reads for each individual cytosine. Therefore, a two-tailed Fisher’s Exact Test was first used to test the “C” and “T” counts for each cytosine between two groups. Then, the differential modification regions (DMRs) were identified across each two samples based on strict criteria as follows: the length of two neighboring CpG sites ≤ 300 bp, the number of CpGs ≥ 5 in a candidate DMR (*P* value < 0.05), and each CpG sites have the same methylated tendency, the number of prominent difference CpG sites ≥ 3. For each of above candidate DMRs, a Fisher’s exact test was performed again based on the mean “C” and “T” counts for all the CpG sites within the candidate DMRs. Besides, a false discovery rate (FDR) adjustment was then performed by the R package of “P.adjust” which is based on BH method (Benjamini and Hochberg 1995). Enrichment analysis of DMRGs was based on molecular functions using Gene ontology web server (http://www.geneontology.org/).

### Validation by bisulfite sequencing PCR with the conventional Sanger sequencing through Illumina Hiseq2000 analyzer (Hiseq-BSP)

The PCR primers were designed using the online MethPrimer software (http://www.urogene.org/methprimer/index.html). Genomic DNA extractions of IUGR/NBW 5–10 were performed using the same method as mentioned above. 400 ng of DNA samples were converted using ZYMO EZ DNA Methylation-Gold Kit^™^ (ZYMO) and one-third of the elution products were used as templates. PCR amplification was carried out with a thermal cycling program of 94 °C for 1 min, 30 cycles of 94 °C for 10 s, 58 °C for 30 s, 72 °C for 30 s, and then final 5 min incubation at 72 °C. PCR products were purified using the QIAquick Gel Extraction Kit (QIAGEN) and subcloned. Twenty-four colonies from each product were sequenced using the 3730 Genetic Analyzer (Applied Biosystems). Then, the reads were post-processed and aligned to the pig reference regions (all PCR regions) using SOAP aligner (Version 2.01) after sequencing according to a previously published method with default parameters that excluded reads with more than five mismatched bases. Multiple reads mapping to the same position were counted only once to remove potential bias from PCR.

### Association analysis of DMRGs and IUGR-related proteins

Association analysis of the selected DMRGs and IUGR-related proteins was performed using the BioGrid server (https://thebiogrid.org/). The schematic was displayed through Cytoscape software based on the results of all the associated relationship among the selected DMRGs and IUGR-related proteins.

## Results

### Increased sulfur amino acids metabolism in the IUGR pig model

**Fig. 1:**
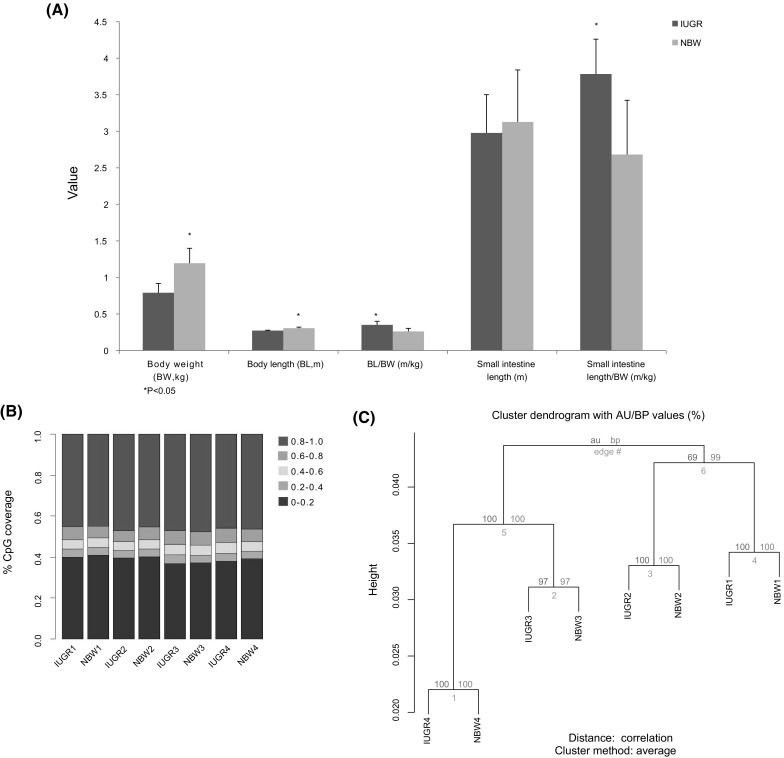
Physiological index of IUGR models compared to NBW littermates and the global methylation levels of all the IUGR and NBW samples. (**a**) Body weight and organ index in IUGR and NBW littermate piglets. (**b**) Global CpG methylation levels of all the eight IUGR and NBW piglets. The methylation levels of all CpGs patterns in the eight piglets were calculated and categorized into five color-coded states. CpG coverage (y-axis) shows the proportion of CpGs covered with different methylation levels. (**c**) Clustering based on methylation of CpGs in the whole genome. The “pvclust” tree diagram clustering is based on CpGs methylation in the whole genome of all the eight samples, and it shows that the methylation levels are diverse among the 4 pairs of IUGR and NBW piglets from different gilts

The IUGR pig model was established using the same protocol introduced in our previous study (Che et al. 2010). We selected eight male piglets from four litters, half of which were IUGR, and the other half were NBW ones (S1 Table). Piglets from IUGR and NBW groups showed significantly different body weight and length (*P* value < 0.05), indicating for clearly restricted growth of IUGR piglets. Particularly, more than 40% of the body weight loss was observed in IUGR piglets (0.94 ± 0.11 kg) in comparison with NBW littermates (1.56 ± 0.24 kg). Accordingly, the relative body length and small intestinal length were higher in IUGR than NBW littermates (*P* value < 0.05) (S1 Table, Fig. 1a).

The plasma concentrations of cysteine, leucine, arginine, and lysine tended to be increased (*P* = 0.06–0.08) in IUGR relative to their NBW littermates (Table 1). Besides, the IUGR piglets had markedly higher concentration of plasma urea (651.47 ± 183.08 µM, *P* value < 0.05) than NBW (340.13 ± 83.69 µM), which suggested that there was abnormal protein metabolism in IUGR piglets. Considering the potential role of sulfur amino acid metabolism in DNA methylation and fetal programming, we further analyzed the mRNA expression of enzymes related to sulfur amino acid metabolism, this result indicated that methionine synthase (MS) was 6.5-fold increase (*P* value < 0.01) in the jejunum tissue of IUGR relative to normal littermates (S3 Table).

**Table 1:** Plasma concentrations of amino acids and urea in IUGR and NBW littermate piglets

	IUGR	NBW
Sulfur metabolism amino acids, µM		
Methionine	19.33 ± 6.27	8.96 ± 1.91
Taurine	18.61 ± 1.24	24.55 ± 3.79
Serine	51.01 ± 7.38	51.97 ± 9.62
Glycine	88.60 ± 6.45	98.40 ± 9.40
Cystathionine	9.50 ± 1.31	7.89 ± 0.74
Cysteine	20.87 ± 3.31	13.59 ± 3.76
Indispensable amino acids, µM		
Arginine	55.87 ± 11.93	28.85 ± 6.80
Histidine	20.19 ± 4.52	15.43 ± 1.73
Isoleucine	26.22 ± 4.40	23.86 ± 2.92
Leucine	57.26 ± 9.26^a^	31.34 ± 4.61
Lysine	101.44 ± 20.85	54.59 ± 11. 90
Phenylalanine	36.56 ± 3.80	28.05 ± 6.00
Threonine	42.06 ± 6.93	41.49 ± 9.89
Dispensable amino acids, µM		
Alanine	82.27 ± 16.52	108.17 ± 13.80
Aspartate	4.71 ± 1.28	4.57 ± 1.13
Citrulline	44.71 ± 7.46	38.06 ± 2.53
Glutamate	27.35 ± 6.37	25.36 ± 2.64
Hydroxyproline	11.36 ± 2.01	21.36 ± 4.37
Ornithine	32.11 ± 5.61	25.05 ± 4.26
Proline	96.80 ± 6.62	106.56 ± 9.00
Tyrosine	46.98 ± 8.42	52.67 ± 9.57
Urea, µM	651.47 ± 183.08^a^	340.13 ± 83.69

Results are mean ± SEM (*n* = 4 per group)

^a^*P* value < 0.05 (within a row, values with different superscript letters mean significant differences.)

### Genome-wide DNA methylation profiling of IUGR piglet intestines

Based on the diverse intestinal sulfur amino acid metabolism in IUGR and NBW piglets, we expected that there might be different DNA methylation levels between the jejunum tissues of IUGR and their NBW littermates. The RRBS technology was applied to generate high resolution of DNA methylomes for jejunum tissues from IUGR piglets and their NBW littermates. A theoretical number of 2.2 million distinct CpG dinucleotides could be sequenced by RRBS (Gao et al. 2014), which covered nearly half of CpG dinucleotides (~ 1 million) in the promoters/CpG islands in pig genome (S1 Fig). We selected the genomic DNA of piglets from the same litters, thereby established 4 pairs of RRBS libraries from the four litters (pair 1–4) of IUGR and their NBW littermates (see “Materials and methods”). The total raw reads of these samples were about 93.99–137.52 million, corresponding to the unique mapping rate of 71.02–94.77%. As a result, a total of 43.1 gigabases (Gb) clean DNA sequencing reads were gained with at least 4.6 Gb for each library (S4 Table), with the average bisulfite conversation rate of 98.10%. The sequencing reads reached an average depth of at least 15.6 per strand in these RRBS libraries and covered 89% of the predicted non-repetitive *Msp* I fragments (S2 Fig). This guaranteed a good coverage of genomic CpG loci that were represented in RRBS library.

Consistent with the previous observations of mammalian DNA methylomes, we found that the methylated non-CpG sites were rare (< 1%). To adjust the false-positive rate (incomplete bisulfite conversion and sequencing error), we used the rate of unconverted non-CpG cytosines (non-CpG methylation level) as a background to calibrate the methylated CpG identification by a method based on binomial test that was described by Lister et al. (Lister et al. 2009). After calibration, from 3.1 to 4.3 million mCs of the 8 samples were counted, in which the majority (~ 96%) were located in CpG contexts. For subsequent analysis, we required at least 4 reads’ depth to determine a methylated CpG in one sample. This threshold covered an average of 76% of the CpGs in the *Msp* I fragments (S2 Fig). Using these qualified CpG sites, we first characterized the eight DNA methylomes of IUGR piglets and their NBW littermates. The individual CpG sites were categorized into quintiles based on their DNA methylation levels, as shown in Fig. 1b, to infer the global pattern of DNA methylomes. There were no notable differences in global DNA methylation of IUGR piglets compared with NBW littermates. Furthermore, clustering analysis based on the CpG methylation revealed greater differences among these four litters of piglets rather than differences between each IUGR piglet and its paired NBW littermate (Fig. 1c). This also suggested that global DNA methylation was not altered observably in IUGR piglets compared to their NBW littermates.

### Regional methylation defects mainly located on chromosome X in IUGR piglets

**Fig. 2:**
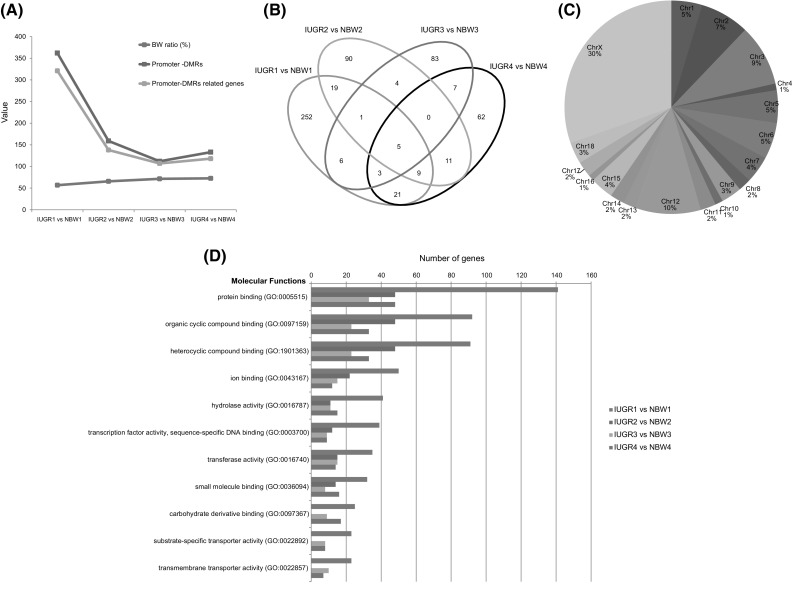
Summary of promoter-DMRs induced by IUGR. (**a**) Smaller IUGR piglets represent more changes in DNA methylation. The tendency of body weight (BW) ratio, promoter-DMRs and promoter-DMRs related genes are presented by line chart in three kinds of fold lines. The fold lines reflect that the lowest BW ratio is corresponding to the largest amount of DMRs. (**b**) Venn diagram of promoter-DMRs related genes in the four pairs of IUGR and NBW piglets. It shows the result of the cross-matching genes with DMRs overlapping with CpG island promoters with respect to the IUGR and NBW pairs. (**c**) Pie chart represents the average distributions of DMRs from the IUGR and NBW pair 1~4 in all the chromosomes. It is indicated that most of the DMRs are located on chromosome X. (**d**) Enrichment analysis result of biological processes for the promoter-DMR genes in the 4 pairs. The x-axis indicates the number of genes, and the y-axis indicates different biological processes. The bar chart shows the different number of genes involved biological processes from the four pairs of IUGR and NBW piglets

Then, the pair-wise comparisons between IUGR piglets and NBW littermates from the same gilts were carried out to screen DMRs based on a strict criteria (see “Materials and methods”). As a result, on average 2831 DMRs were identified between each pair of IUGR and NBW piglets, as summarized in S6 Table. Consistently more CpGs were revealed in the IUGR versus NBW pair of piglets that contain more DMRs in the genome, as indicated by measuring the ratio of CpGs located in DMRs to the total CpGs in the genome. Moreover, a larger number of DMRs were correlated with lower ratio of body weights between IUGR and NBW piglets that were born from the same litter (Fig. 2a), suggesting a considerable link between IUGR and intestinal methylomes.

We then characterized the distribution of DMRs and found that these DMRs occurred more often in intergenic regions and gene bodies, rather than in promoters, despite of which, a number of DMRs were also frequently revealed in CpG islands (CGIs) (Fig S4). For the important role of CGIs in the regulation of gene expressions, the transcription of genes containing DMR in CGIs located within their promoters could be affected. Interestingly, it was observed that most of DMRs in promoters of genes located on chromosome X, more frequently than on other chromosomes (Fig. 2c). Since the samples used in this research for DNA methylation analysis were all male ones, this enrichment of DMRs on chromosome X was not due to the X-chromosome inactivation of female genomes. Thereby, our results might suggest that a series of genes affected by IUGR were located in chromosome X. Enrichment analysis of DMR-related genes (DMRGs) by Gene ontology (GO) server based on molecular function indicated that majority of these genes encoded binding proteins that may function as key regulatory factors, such as transcription factors or signaling proteins (Fig. 2d).

### Key DMRGs were consistently validated in extra IUGR and NBW piglets

**Fig. 3:**
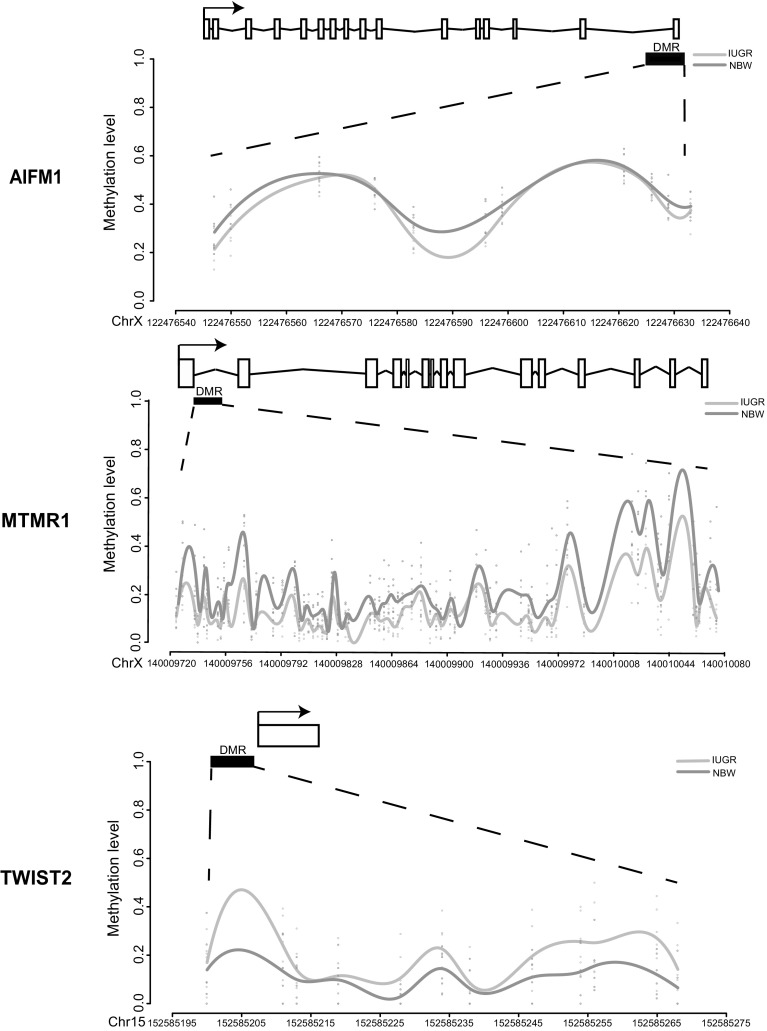
Validation of DMR related genes (DMRGs) by Hiseq-BSP. This is combined with Hiseq-BSP in another 6 pairs of IUGR and NBW piglets (pair 5~10) for methylation validation of the selected 19 DMRGs according to RRBS analysis result. Three genes (AIFM1, MTMR1, and TWIST2) were successfully validated and showed the same methylation level and overlapped regions of DMR with the RRBS analysis results. TWIST2 gene was hypermethylated in its promoter (left) in IUGR piglets, while the AIFM1 and MTMR1genes were hypomethylated in their promoter in IUGR piglets

To validate the candidate genes with differential DNA methylation, we selected 19 genes with promoter DMRs that repeatedly revealed in at least three pairs of IUGR and NBW piglets (S8 Table). Most of these DMRGs were located on chromosome X and had higher methylation levels in NBW piglets compared with IUGR littermates, except for twist homolog 2 (TWIST2), which was on chromosome 15 with increased methylation level in IUGR piglets. We then performed bisulfite sequencing PCR combined with HiSeq sequencing (HiSeq-BSP) (Gao et al. 2014; Mensaert et al. 2014) and nine genes (MTMR1, HAUS7, FAM127C, AIFM1, PIM2, TWIST2, IRAK1, BCAP31, and SOX3) were successfully cloned to validate these DMRs in another six pairs of piglets (IUGR/NBW pair 5–10). Considering that the DMRs may shift among different individuals, we re-performed pair-wise comparisons to search for DMRs from the amplified PCR fragments among each pair. As a result, three out of the nine genes were successfully validated to have the same regions and methylation tendency with DMRs from the pair-wise comparison among IUGR/NBW1-4, including TWIST2, AIFM1, and MTMR1 (S10 Table). The DMRs found in these three genes were all located in or near their promoter regions, potentially affecting their gene expression (Fig. 3). Among these genes, TWIST2 was consistently observed with hypermethylation in its promoter DMR in IUGR piglets. As TWIST2 functions in regulating immune-metabolic genes (Galván et al. 2015; Mudry et al. 2015; Zheng et al. 2015), its potential down-regulation of transcription due to promoter hypermethylation may hamper intestinal development in IUGR piglets. The other two genes, AIFM1 and MTMR1, were both hypomethylated in the IUGR piglets, implying for potential up-regulation of gene expression. AIFM1 gene encodes mitochondrial apoptosis-inducing factor (AIF) that is critically important for energy metabolism and execution of the caspase-independent cell death pathway (Sevrioukova 2016). MTMR1 gene is a phosphatase which represents a novel target for abnormal mRNA splicing in myotonic dystrophy, and its abnormal expression is proved to be related to impair muscle differentiation (Santoro et al. 2010). Therefore, the abnormal up-regulated gene expression due to hypomethylation of these two genes might be closely related to the symptoms caused by IUGR.

### Network analyses revealing DMRGs involved in pathways of innate immunity and apoptosis

**Fig. 4:**
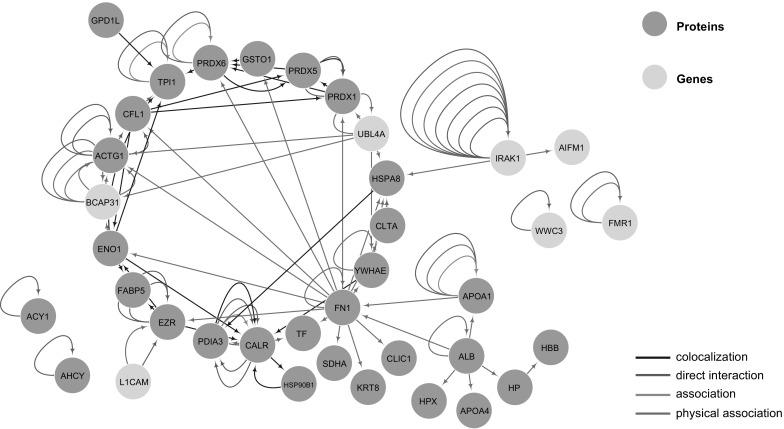
Correlation networks of DMRGs and the IUGR related proteins. Association analysis among DMRGs and the IUGR related proteins is shown in this network. The yellow circles present DMRGs and the purple ones are proteins. Lines with different colors indicate various kinds of relationship between these DMRGs and these proteins. It was clear that BCAP31 had direct interaction with ACTG1, while IRAK1 showed physical associations with HSPA8 and AIFM1 separately

The previous studies have confirmed for altered protein expression profiles of small intestines related to IUGR fetuses, including key proteins required for cell structure maintenance and nutrients metabolism (He et al. 2011; Wang et al. 2014), which could contribute to impaired growth and jejunal function. As DNA methylation plays an important role for transcription regulation, it is expected that divergent DNA methylation will ultimately affect protein expression, either directly or indirectly. Since majority of our DMRGs encode binding proteins, we would expect that these DMRG-encoded proteins could interact with a cascade of another proteins so as to affect cellular functions. By integrating the proteome data from one previous study that established the identical IUGR piglet model and identified a serial of proteins related to impairments of intestinal functions (Wang et al. 2010), we inferred the potential protein–protein interaction network among DMRG-encoded proteins and the divergently expressed proteins in IUGR piglet model. BioGrid (https://thebiogrid.org/) database was used for annotation of all the proteins associated with the 19 selected DMRGs overlapped in at least three IUGR and NBW pairs and their potential interactions with the divergent proteins acquired from the proteome data. As a result, we found five DMRG-encoded proteins interacted with a number of divergent proteins, either directly or indirectly (Fig. 4). Among the five DMRG-encoded proteins, IRAK1 interacts with HSPA8 (HSP70), together, they serve crucial roles in Toll-like receptors (TLRs) signaling pathway of innate immunity by mediating the activation of macrophages through microbial pathogens. When the level of maintenance provided by the Hsp90-Cdc37 chaperone module becomes insufficient to maintain IRAK-1 in a stable and functional conformation, it would associate with Hsp70 and ultimately be degraded by the proteasome. This in turn would limit the capacity of TLRs to activate downstream signaling targets of IRAK1 and dampen the inflammatory response of the macrophage (De Nardo et al. 2005). Furthermore, IRAK1 can interact with AIFM1, together, they are involved in the subnetwork of apoptosis-associated interacting proteins (So et al. 2015). Another protein, BCAP31, can directly interact with ACTG1 (γ-actin) and may have functions in the structural organization of the cytoplasm or contribute to extranuclear events, such as membrane remodeling, during the execution phase of apoptosis. In summary, the DMRGs verified in IUGR piglets may participate in several pathways related to cell immunity and apoptosis.

## Discussion

The previous studies have shown that IUGR not only predisposed to postnatal metabolic disease (Horvath et al. 2013; Liu et al. 2014), but also exhibited poor intestinal development and function in neonatal period. The biochemical, genomic, and proteomic analyses on intestinal tissues were performed to study the underlying mechanisms of the gastrointestinal dysfunction in IUGR (Fança-Berthon et al. 2009; D’Inca et al. 2011; He et al. 2011); however, the epigenetic regulation in inducing the transcriptional changes is still unknown. In the present study, considering the essential role of epigenetic modification, especially DNA methylation in transcriptional regulation, we performed the very first study to screen the DNA methylation differences of intestine between IUGR and NBW littermates. Through DNA methylation analysis of jejunum tissues from four pairs of IUGR and NBW piglets, we found that IUGR could lead to DNA methylation changes at different levels, and the key genes (BCAP31, AIFM1, and IRAK1) regulated had direct associations with IUGR-related proteins. These findings suggested a great possibility of DNA methylation changes involving in intestinal development and function of IUGR offspring, which could supply more information on the intestinal dysfunction of IUGR neonates.

In this study, we used pig as animal model for the anatomic, physiological, as well as genetic similarities between pigs and humans (Bendixen et al. 2010). Particularly, pig has been recognized as a proper animal model to study intestinal development or diseases (Sangild 2006), and pigs exhibit the most severe naturally occurring IUGR among domestic animals. In our study, the significantly lower birth weight and higher organ index in IUGR pigs was consistent with the previous IUGR pig model (D’Inca et al. 2011). In addition, we found that there was increasing plasma concentration of cysteine and markedly higher methionine synthase mRNA expression level in the jejunum of IUGR piglets. Methionine synthase is involved in the re-methylation of homocysteine to methionine; the markedly increased methionine synthase expression is considered to compensate for the hyperhomocysteinemia, which is closely linked to IUGR (de la Calle et al. 2003). Furthermore, the abnormal metabolism of sulfur amino acids in IUGR pigs might disturb the production of S-adeonsylmethionine (SAM), a primary methyl donor for DNA methylation (Anderson et al. 2012), which had possibility in playing a vital of important role in inducing epigenetic changes of IUGR piglets. Despite that there were no global-scale differences of DNA methylation between the IUGR and NBW piglets, divergently methylated regions were revealed. Relative to NBW littermates, we found that lower body weight of IUGR piglets was corresponding to more DMRs in the intestine, suggesting that the global intestinal epigenome dysfunction might be linked to the degree of IUGR impairment and relatively unstable methylation pattern.

Moreover, the enrichment of DMRs in promoters on chromosome X indicated a potential link of epigenetic dysfunction on chromosome X to IUGR development. This might explain the reasons that there was a gender-specific developmental pattern of IUGR pigs, in which the male pigs could not exhibit postnatal catch-up growth, but female IUGR pigs achieved similar development as control counterparts (Gonzalez-Bulnes et al. 2012). The similar results by gender-related effects on catch-up growth were also described in laboratory rodents (Oyhenart et al. 2003) and human-beings (Amador-Licona et al. 2007). Moreover, the hepatic IGF-I histone code was found to be abnormal in the male rat (Fu et al. 2009). These results together indicated that chromosome X seemed to play an important role in the development of IUGR and postnatal metabolism syndrome.

Interestingly, we found that the DMRs among four pairs of IUGR versus NBW comparisons were limited, suggesting that the alterations of DNA methylation during IUGR was a random process. This result might be attributed to the potential genotypic background, uncertain maternal factors. However, most of the DMRGs from the four pairs of IUGR and NBW piglets were indicated to encode binding proteins through enrichment analysis, suggesting for their main regulatory roles. Then 19 common DMRGs repeated in at least three pairs were selected and nine genes were successfully validated using an extra six pairs of IUGR and NBW piglets. Three genes (AIFM1, MTMR1, and TWIST2) were validated as key candidate genes by Hiseq-BSP. The TWIST2 gene, located on chromosome 15, has been involved in the hypermethylation of tumor stroma (Galván et al. 2015); therefore, the hypermethylation-induced lower expression of TWIST2 might affect the intestinal development and function. On the other side, another two genes with hypomethylation were located on chromosome X. The protein encoded by AIFM1 gene is important for energy metabolism and execution of the caspase-independent cell death pathway (Sevrioukova 2016), while the MTMR1 gene encoded a phosphatase (Bong et al. 2016) that is related to muscle differentiation (Buj-Bello et al. 2002; Santoro et al. 2010). Therefore, the hypomethylation-induced up-regulation on the expressions of two genes could affect the body development and metabolism, which was in accordance with the disease symptoms caused by IUGR and might elicit long-term complications.

Association analysis of DMRGs and IUGR-related proteins revealed that there were three main genes (BCAP31, AIFM1, and IRAK1) involved in the correlation networks. The BCAP31 gene encoded B-cell receptor-associated protein 31, as a chaperone, its specific interaction with ACTG1 (γ-actin) may have functions in the structural organization of the cytoplasm or contribute to extranuclear events, such as membrane remodeling, during the execution phase of apoptosis (Ducret et al. 2003). IUGR could lead to the impairments of body development and metabolism; the occurrences of cell apoptosis might be a stress response induced by IUGR. Besides, in the physical interaction between IRAK1 and HSPA8 (HSP70), they were both related to TLRs playing a crucial role in innate immunity. As IRAK1 was a key component of TLR signaling pathways, its association with HSP70 would in turn limit the capacity of TLRs to activate downstream signaling targets of IRAK1 and dampen the inflammatory response of the macrophage (De Nardo et al. 2005). On the other side, as to the physical interaction of IRAK1 and AIFM1, it was proved to be related with the tumor necrosis factor-related apoptosis-inducing ligand (TRAIL) (So et al. 2015). Considering of the close association between IRAK1 and the immunity system, its hypomethylation in IUGR piglets may explain the impairments of body development and related metabolism. Based on these gene functions and their close associations with cell apoptosis and immunity, DNA methylation alteration caused by IUGR would absolutely affect a series of related biological processes and bring about disease symptoms during the period of body development and metabolism.

In conclusion, IUGR, as an important cause of morbidity and mortality in fetuses and neonates, could lead to abnormal intestinal DNA methylation in the pig model. This alteration of DNA methylation would affect the body development and metabolism through the regulation of related genes expressions and their functions in various biological processes, such as cell apoptosis, cell differentiation, and immunity. That may provide clues on the intestinal dysfunction of IUGR neonates and point prospective directions on the linkage of maternal environment, including nutrition, medicine, and other environmental factors, to the offspring diseases and other postnatal complications. Since the crucial role of innate immunity in the body development and health of neonates, it is very necessary to explore more on its association with epigenetic regulation and neonatal diseases like IUGR.

## Electronic supplementary material

Below is the link to the electronic supplementary material.

Supplementary material 1 (DOC 48 KB)

Supplementary material 2 (PDF 504 KB)

Supplementary material 3 (XLSX 313 KB)
