# Supplementary material for: Genome-wide DNA methylation analysis in jejunum of Sus scrofa with intrauterine growth restriction
Source: Mol Genet Genomics. 2018 Feb 1;293(4):807–18. doi: 10.1007/s00438-018-1422-9 (PMC6061055; doi:10.1007/s00438-018-1422-9)
Supplement: Supplementary file 1 — Supplementary material 1 (DOC 48 KB) [file 438_2018_1422_MOESM1_ESM.doc]

**Supporting Information**

**S1 Figure The *Msp* I based reduced representation.**

The *Msp* I based reduced representation fraction contains ~7.9% of all CpGs in the pig genome, but is significantly enriched for promoter/CpG islands.

**S2 Figure Percent of CpGs covered by read depth thresholds.**

It is based on CpG sites from restriction digestion fragments of 40~220 bp. Values are means ± SD (n = 4).

**S3 Figure DNA methylation levels with genomic features.**

The Pearson’s correlation between CpG methylation level and features of pig chromosomes (n=20). The CpG methylation level (%) was plotted against the length, GC content, CpG o/e ratio, repeat density, gene density, and CpG density of individual chromosome. Line represents linear regression. Values are means ± SD (n = 4).

**S4 Figure Distribution of DMRs.**

It was shown the percentage of CpGs within DMRs in different genomic elements.

**Supplementary file**

This file includes all the supplemental tables (S1 Table~S11 Table) in the manuscript.
