# Supplementary figures and images for: Genome-wide DNA methylation analysis in jejunum of Sus scrofa with intrauterine growth restriction
Source: Mol Genet Genomics. 2018 Feb 1;293(4):807–18. doi: 10.1007/s00438-018-1422-9 (PMC6061055; doi:10.1007/s00438-018-1422-9)

S1 Figure

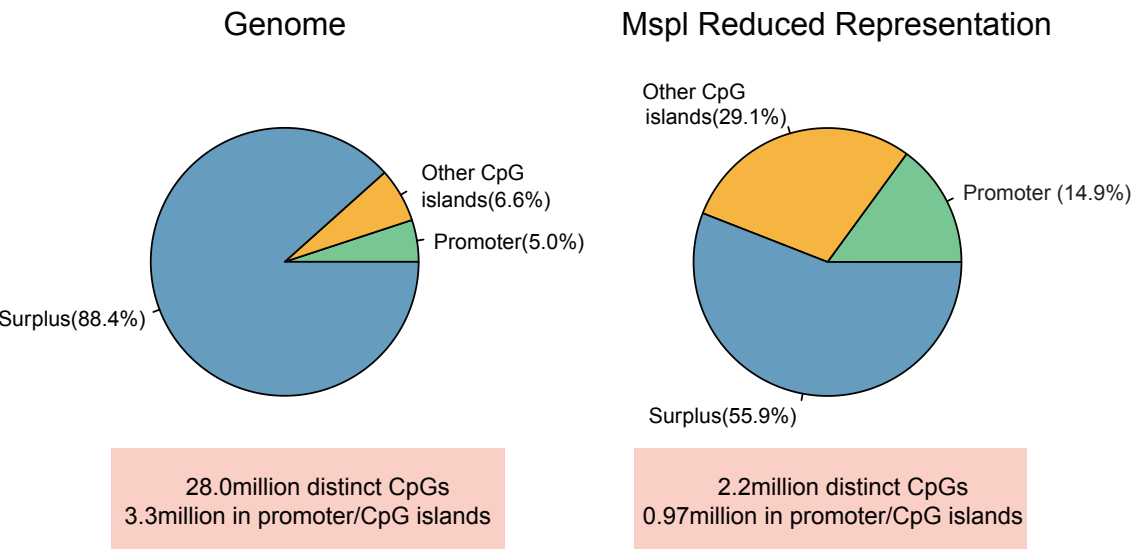

S2 Figure

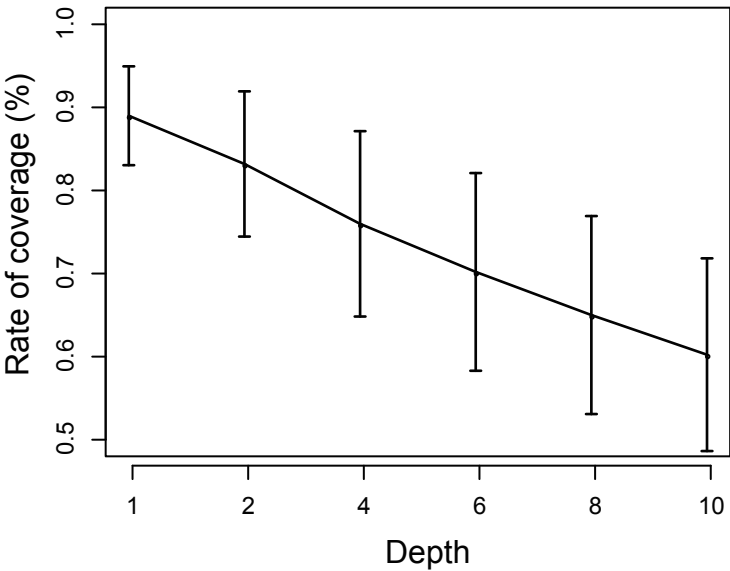

S3 Figure

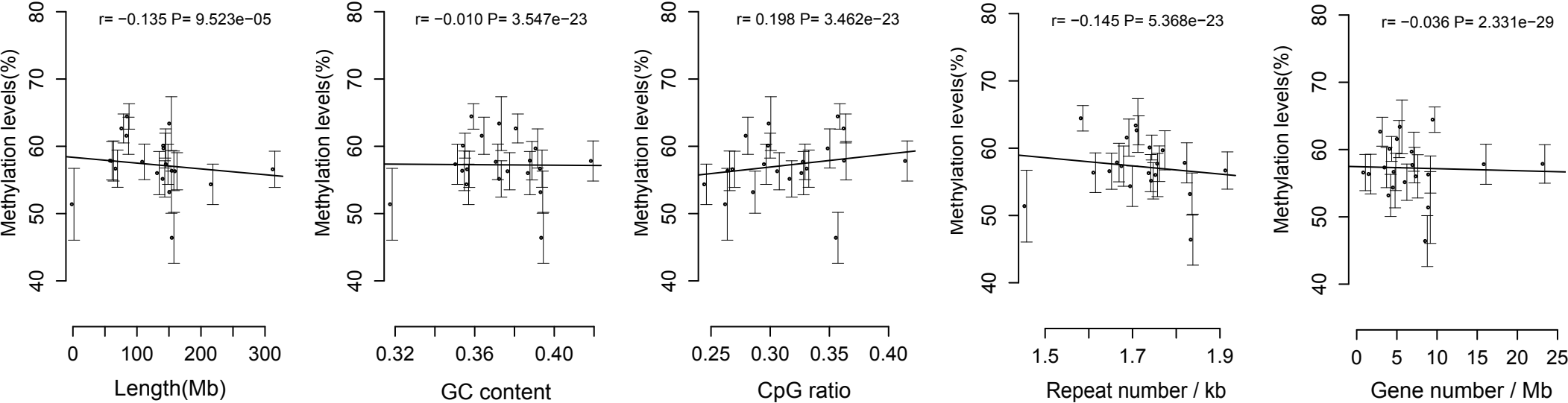

S4 Fig

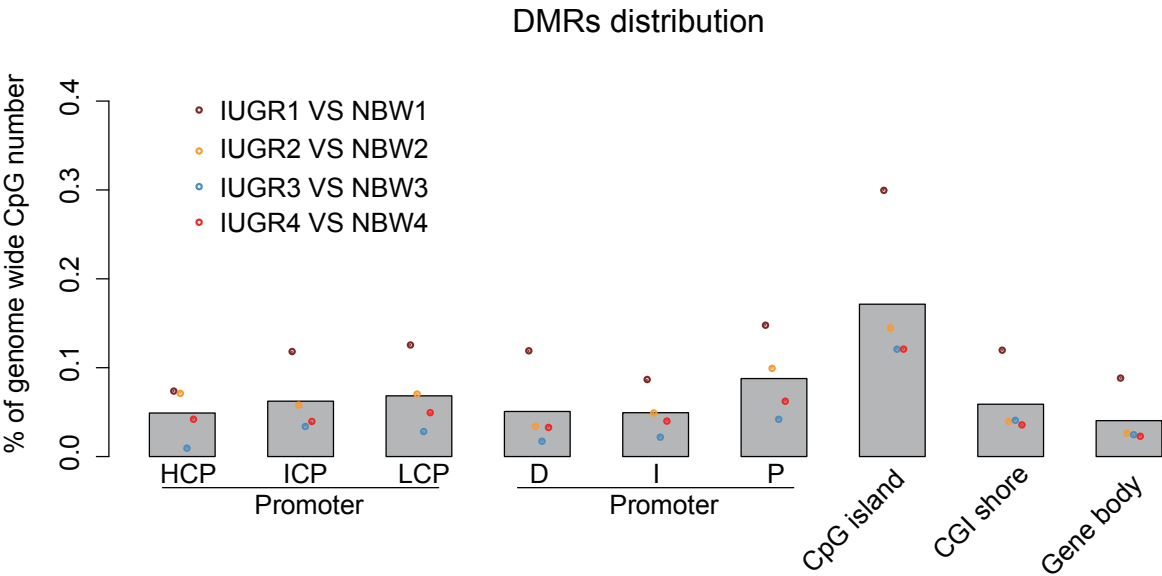

Supplement: Supplementary file 2 — Supplementary material 2 (PDF 504 KB) [file 438_2018_1422_MOESM2_ESM.pdf]
